# A performance predictor of beamforming versus time-reversal based far-field wireless power transfer from linear array

**DOI:** 10.1038/s41598-021-02244-9

**Published:** 2021-11-23

**Authors:** Hong Soo Park, Sun K. Hong

**Affiliations:** School of Electronic Engineering, Soongsil University, Seoul, 06978 Republic of Korea

**Keywords:** Engineering, Electrical and electronic engineering

## Abstract

For far-field wireless power transfer (WPT) in a complex propagation environment, a time-reversal (TR) based WPT that can overcome the drawbacks of conventional beamforming (BF) by taking advantage of multipath has been recently proposed. However, due to the WPT performance of BF and TR depending on the complexity of the propagation environment, the performance prediction between BF versus TR would be required. We present a detailed and generalized analysis of the recently proposed performance metric referred to as the *peak received power ratio* (PRPR) for linear array-based WPT. Here, the effectiveness of *PRPR* is verified via measurement for free space and indoor scenarios. The results demonstrate that *PRPR* is directly related to the complexity of the propagation environment and the corresponding power transmission capability of BF and TR. That is, the higher the complexity, the greater the value of *PRPR* and TR outperforms BF with higher peak power given the same average transmit power and vice versa. The mode decision between BF and TR based on *PRPR* potentially promises efficient far-field WPT even in a dynamic propagation environment.

## Introduction

In recent years, far-field wireless power transfer (WPT) has received considerable interest for various applications in that it can improve battery problems by wirelessly powering small, low-power devices^1–5^. In the conventional far-field WPT systems, array-based beamforming (BF) is commonly used to deliver electromagnetic energy by generating the main beam through an antenna array^6–11^. While various array-based BF techniques have been proposed, their reported performances were mainly based in free space environments. However, in a complex propagation environment where multiple scatterers and reflectors exist (e.g. indoor), there is a potential limitation of BF that the beam generated by the array could be impaired due to multipath, which makes effective wireless power transmission to a desired location unfeasible.

To overcome such disadvantages of BF, time-reversal (TR)-based far-field WPT has been recently proposed^12–15^. By inherently taking advantage of multipath in a complex propagation environment, TR allows spatial and temporal wave focusing and thereby enabling selective transmission of electromagnetic power at the receiver^13–22^. It is demonstrated that sending TR waveforms can deliver higher peak power at desired locations compared to sending narrowband signals (CWs) even with a single transmit antenna^14,15,21^. Furthermore, in our recent study, it is also shown that TR-based WPT from an antenna array can outperform array-based BF given the same average transmit power in a complex environment^22^. Therefore, previous studies have demonstrated the benefits of TR-based WPT compared to BF in a complex propagation environment.

However, since the power transmission performance between BF versus TR varies depending on the degree of complexity in a given propagation environment, it would be required to selectively utilize BF and TR accordingly. In this regard, we have recently introduced the concept of a performance predictor for linear array-based BF versus TR WPT by defining a term referred to as the *peak received power ratio* (PRPR)^22^. Through theoretical derivation, it was shown that *PRPR* can be used as a performance metric of BF versus TR WPT by simply using the transfer function between the linear transmit array and receive antenna.

In this work, we provide a more detailed and generalized analysis of *PRPR* and demonstrate the feasibility of performance prediction for linear array-based BF versus TR WPT via measurement. The proposed performance metric would provide a reasonable basis for adaptively deciding between BF and TR in a given environment, thereby increasing the efficiency of linear array-based WPT regardless of the propagation environment. In other words, given a WPT system that can utilize both BF and TR, *PRPR* would serve as a core deciding factor for mode selection between BF and TR to achieve optimal wireless power transmission even in a dynamic propagation environment.

## Peak received power ratio based on linear array

**Figure 1:**
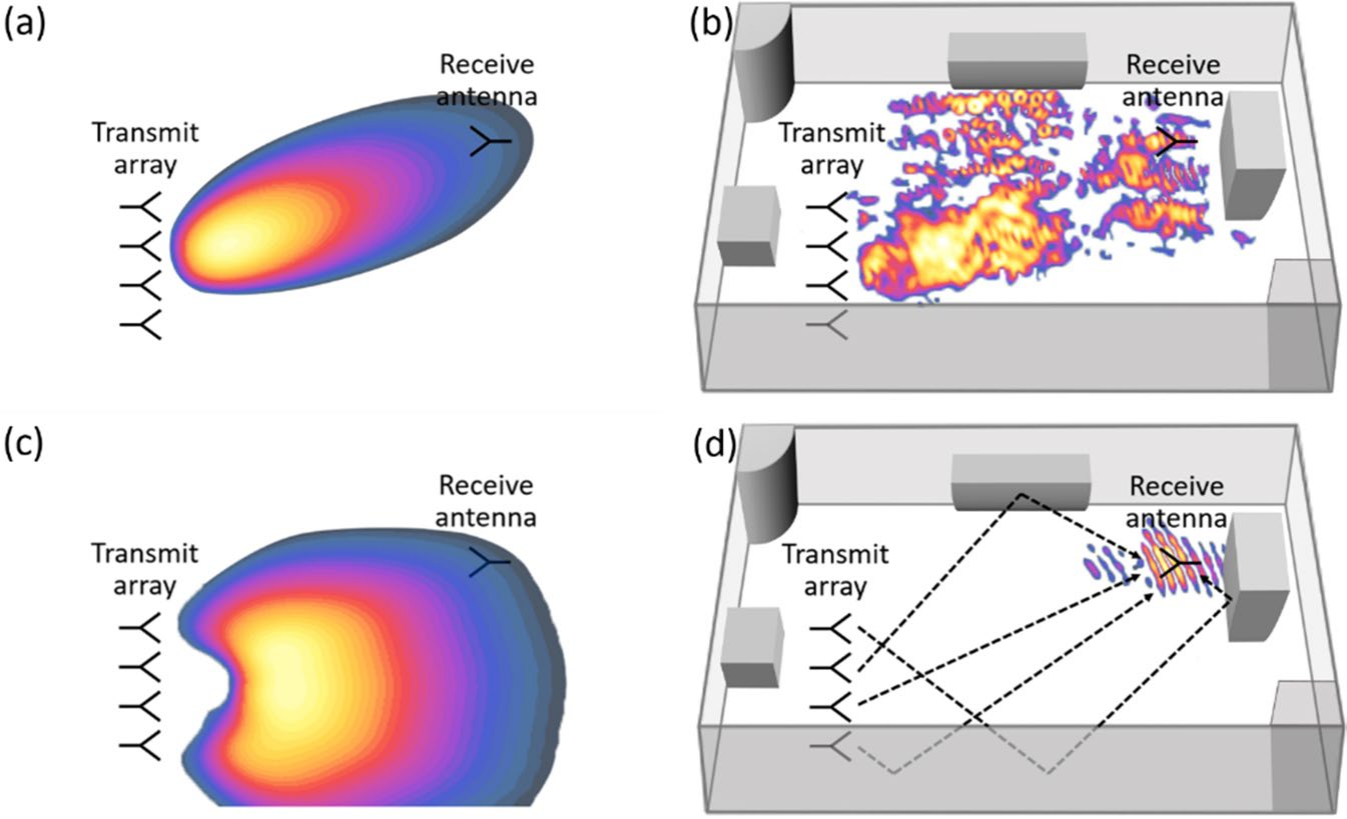
Illustration of array-based far-field wireless power transfer. (**a**) BF in free space. (**b**) BF in an indoor environment. (**c**) TR-based WPT in free space. (**d**) TR-based WPT in an indoor environment.

Figure 1 illustrates the mechanism of the array-based WPT via BF and TR in two representative propagation environments, i.e. free space and indoor scenarios. For BF, the intended beam is properly generated in free space (Fig. 1a), whereas the beam is impaired due to multipath in an indoor environment (Fig. 1b). For TR, wave focusing does not take place in free space when a single transmit antenna is used (Fig. 1c), while selective wave focusing at the location of the receiver is achievable in an indoor environment by taking advantage of multipath (Fig. 1d). Here, we consider an *N*-element linear array and the beacon (pilot) signal is assumed to be a short pulse (containing bandwidth of interest) represented as $p(t)$, where resulting signals of BF and TR are respectively expressed as

1 $$y_{BF} (t) = \sum\limits_{n = 1}^{N} {y_{n,BF} (t)} = \sum\limits_{n = 1}^{N} {A\cos (\omega_{0} t - \phi_{n} ) * h_{n} (t)}$$

and

2 $$y_{n,TR} (t) = B_{n} p(T - t) * [h_{n} (T - t) * h_{n} (t)],$$

where the subscript *n* refers to the *n*th transmit array element, $h_{n} (t)$ is the impulse response between the receiver and *n*th array element, $\omega_{0} = 2\pi f_{0}$ is the center frequency, $\phi_{n}$ is the relative phasing of *n*th array element, *T* is the time duration of the impulse response, and $*$ denotes convolution^22^. Here, *A* and *B*_*n*_ represent the signal amplification. Setting the average transmit power to $P_{in}$ and the input impedance of each array element to $Z_{in}$, *A* and *B*_*n*_ can be expressed as

3 $$A = \sqrt {\frac{{2P_{in} Z_{in} }}{N}}$$

and

4 $$B_{n} = \sqrt {\frac{{P_{in} Z_{in} T}}{{\int\limits_{T} {\left| {p(T - t) * h_{n} (T - t)} \right|^{2} dt} }}} \approx \sqrt {\frac{{P_{in} Z_{in} T}}{{\frac{1}{2\pi }\int\limits_{ - \infty }^{\infty } {\left| {P(\omega )H_{n} (\omega )} \right|^{2} d\omega } }}} ,$$

where $H_{n} (\omega ) = \left| {H_{n} (\omega )} \right|e^{{j\alpha_{n} }}$ is the transfer function between the *n*th array element and receive antenna, and $P(\omega )$ is the Fourier transform of $p(t)$. In Eq. (4), signal amplification *B*_*n*_ for TR can approximately be expressed with the frequency domain terms by Parseval’s relation. Note that the resulting signals are the result of the assumption that only one signal source is used for both BF and TR. That is, based on a single source with the same average transmit power, all array elements transmit simultaneously with a power divider for BF, but only one element transmits at a time in a switching manner for TR. Accordingly, each peak received power is represented using the frequency domain terms as

5 $$\max \{ y_{BF}^{2} (t)\} = \frac{{2P_{in} Z_{in} }}{N}\left| {\sum\limits_{n = 1}^{N} {\left| {H_{n} (\omega_{0} )} \right|e^{{j(\alpha_{n} - \phi_{n} )}} } } \right|^{2}$$

and

6 $$\max \{ y_{n,TR}^{2} (t)\} = \frac{{Z_{in} P_{in} T}}{2\pi }\frac{{\left[ {\int\limits_{ - \infty }^{\infty } {\left| {P^{ * } (\omega )e^{ - j\omega T} \left| {H_{n} (\omega )} \right|^{2} } \right|d\omega } } \right]^{2} }}{{\int\limits_{ - \infty }^{\infty } {\left| {P(\omega )H_{n} (\omega )} \right|^{2} d\omega } }},$$

where $e^{ - j\omega T}$ is associated with a time shift *T* in $p(T - t)$. Consequently, substituting Eqs. (5) and (6), a generalized *PRPR* between BF versus TR of each transmit element can be defined as follows:

7 $$PRPR_{n} = \frac{{\max \{ y_{n,TR}^{2} (t)\} }}{{\max \{ y_{BF}^{2} (t)\} }} = \frac{NT}{{4\pi }}\frac{{\left[ {\int\limits_{ - \infty }^{\infty } {\left| {P^{ * } (\omega )e^{ - j\omega T} \left| {H_{n} (\omega )} \right|^{2} } \right|d\omega } } \right]^{2} }}{{\left| {\sum\limits_{n = 1}^{N} {\left| {H_{n} (\omega_{0} )} \right|e^{{j(\alpha_{n} - \phi_{n} )}} } } \right|^{2} \int\limits_{\infty }^{ - \infty } {\left| {P_{n} (\omega )H_{n} (\omega )} \right|^{2} d\omega } }},\;\;\;\;\;n = 1, \ldots ,N,$$

which implies that *PRPR* can be calculated only using *N*, *T*, beacon signal (short pulse), and the transfer functions. From Eq. (7), *PRPR* greater than 1 means that TR delivers a peak power higher than BF and vice versa. Since the level of *PRPR* depends on the propagation environment and array configuration, a reference value other than 1 may also be used.

By comparing *PRPR* of each array element, it is possible to utilize *PRPR* for more effective TR-based WPT. Since a large *PRPR* value indicates that high peak power is received by TR, it is advantageous to improve the performance of TR-based WPT to use only the element with the largest *PRPR*. In other words, despite the use of a transmit array, using only one element can provide the optimal performance of TR-based WPT. Therefore, using *PRPR* one can not only determine the mode by predicting the performance of BF versus TR in a given propagation environment but also select an optimal array element for TR operation.

## Experimental setup

**Figure 2:**
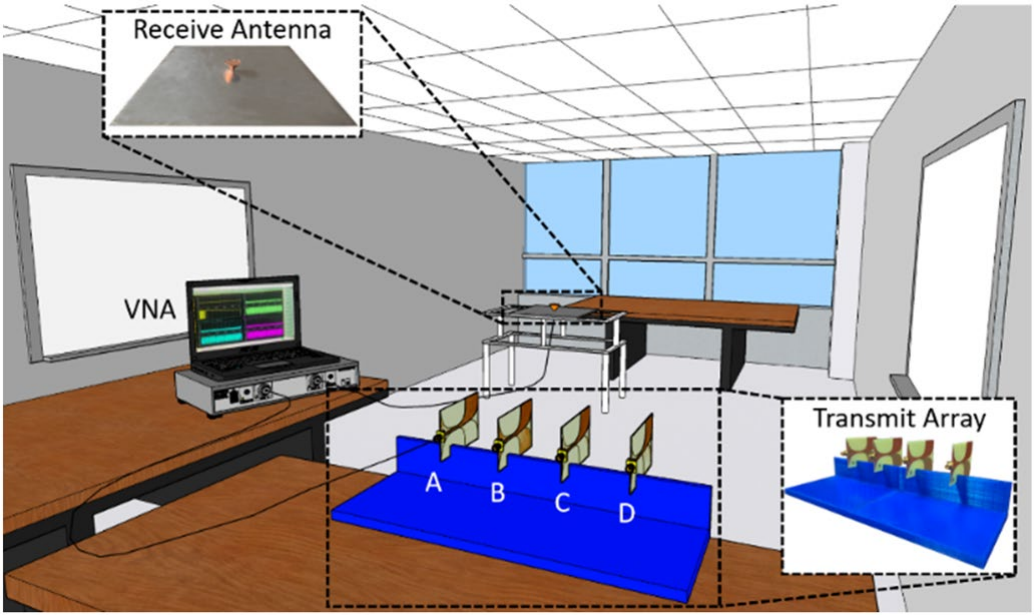
An illustrated view of an office room with the measurement setup.

The relationship between *PRPR* and linear array-based WPT is validated via indoor measurement. The experiment setup consists of an office meeting room (7.35 m × 3.2 m × 2.5 m) furnished with desks and whiteboards, as shown in Fig. 2. Here, Vivaldi antennas and a monoconical antenna, both designed to cover 1.95–2.95 GHz, are used for the transmit array and receive antenna, respectively^23^. Since the choice of antenna is not a major factor for analyzing *PRPR*, which compares peak received power, we use these antennas for their simplicity in design and fabrication. Based on the free space wavelength $\lambda_{0}$ of 12.25 cm at the center frequency $f_{0}$ of 2.45 GHz, the transmit array is constructed using four Vivaldi antennas with a $\lambda_{0} /2$ spacing. For a linear scan to observe the spatial variation of received peak power, the position of the receive antenna is varied laterally at an increment of 0.2 $\lambda_{0}$ (2.45 cm) from the reference position (0 cm) to $\pm 2\lambda_{0}$. From the positions of the receive antenna, the targeted receive locations are set to 11 different locations over the range of $\pm \lambda_{0}$ (± 12.25 cm) at an increment of $0.2\lambda_{0}$(2.45 cm). The reference position is 2.4 m in down range and 0.9 m in cross range from the center of the transmit array.

The transfer function $H_{n} (\omega )$ of one transmit-receive antenna pair is measured at a time over a bandwidth of 1.95–2.95 GHz using an Anritsu MS46122B vector network analyzer (VNA). The measured transfer functions are converted into the time-domain impulse response $h_{n} (t)$, and the received signals for BF and TR, i.e. $y_{BF} (t)$ and $y_{TR} (t)$ are then obtained in the processing. For the processing, beacon signals are assumed to be a Gaussian pulse with a bandwidth of 200 MHz centered at $f_{0}$. Also, the average transmit power is set to 30 dBm for both BF and TR.

**Figure 3:**
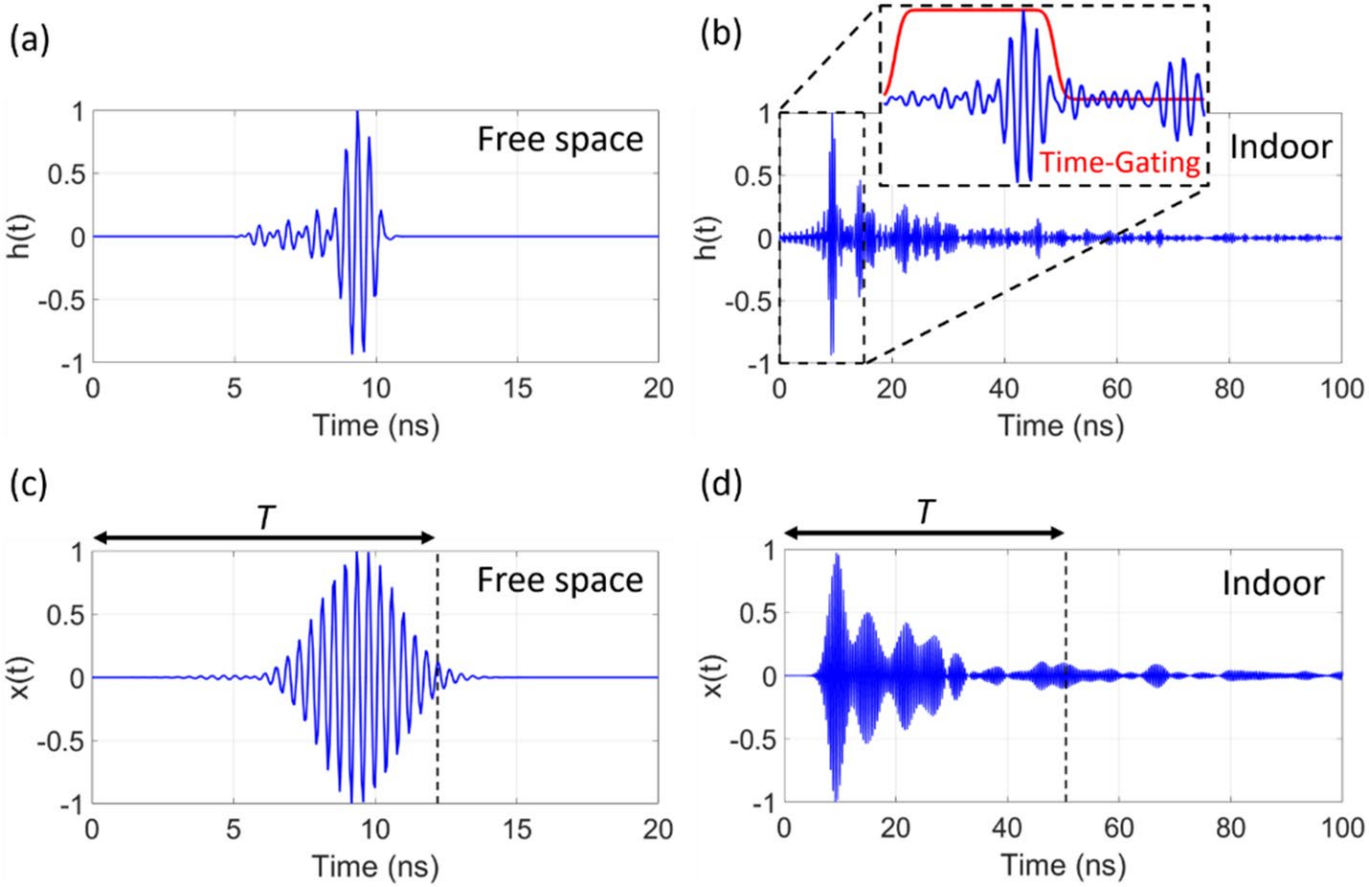
Example impulse response $h\left(t\right)$ and Gaussian-modulated signal *x*$\left(t\right)$ in each propagation environment. (a) $h\left(t\right)$ in free space. (b) $h\left(t\right)$ in an indoor environment. (c) *x*$\left(t\right)$ in free space. (d) *x* $\left(t\right)$ in an indoor environment. *T* refers to a time at which the signal amplitude decays to 10% of the peak value.

To compare *PRPR* and the corresponding power transmission results in a propagation environment with different complexity, we consider the aforementioned two representative propagation environments (i.e. free space and indoor). However, indoor is inherently a multipath environment (e.g. reflecting walls exist), which imposes difficulties in realizing an ideal free space. Hence, we apply time-gating using a smooth rectangular window to the impulse responses $h_{n} (t)$ measured in an indoor environment, which mimics a free space measurement by extracting only the first pulse corresponding to the direct path^24,25^. Figure 3 shows an example of the impulse response and Gaussian-modulated signal for free space and indoor. The impulse response slowly decays due to multipath time-delays for the case of indoor (see Fig. 3b), while only one short pulse appears for free space (see Fig. 3a). To generate the received beacon signal $x(t)$, these impulse responses are modulated with a Gaussian pulse (see Fig. 3c and 3d) and then time-reversed to become the input signal for TR. The time duration *T* is set to a point at which the signal amplitude of $x(t)$ is decreased to 10% of the peak value.

## Results and discussion

### *PRPR* calculation

**Figure 4:**
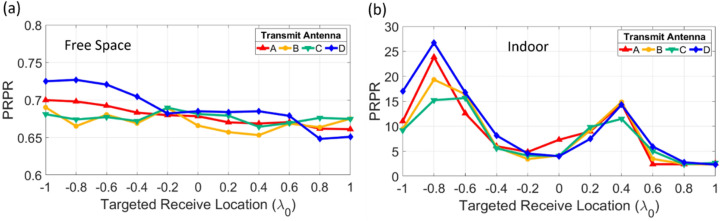
*PRPR* calculated using the measured transfer function between transmit array element and each targeted receive antenna location. (**a**) Free space, (**b**) indoor environment.

From the measured transfer functions between each of the four array elements and the 11 cases of targeted receive locations, *PRPR* is theoretically calculated using Eq. (7), and the resulting values for free space and indoor environment are plotted in Fig. 4. In the case of free space, all *PRPR* values are less than 1, which implies that BF delivers a peak power higher than TR given the same average transmit power. In addition, it can be seen that the *PRPR* values in general are similar for all targeted receive locations and vary slightly depending on the array element (Fig. 4a). This means that regardless of the targeted receive antenna location, BF has a consistent performance with the correct phasing of all array elements, but the performance of TR depends on the fixed position of each array element. That is, the impulse response for TR in free space is a short pulse (beacon signal itself), and the magnitude of the time-reversed short pulse (input signal for TR) transmitted to the receive antenna varies according to each array element.

In the case of an indoor environment, all *PRPR* values are greater than 1, and TR is expected to deliver a peak power higher than BF given the same average transmit power (Fig. 4b). Unlike free space, the indoor results indicate that all array elements have similar *PRPR* values, which vary depending on the targeted receive location. Such a phenomenon is related to the eigenmodes determined by the geometry and boundary conditions of the room. Despite the correct phasing for BF, the beam would be damaged by multipath and dominated by the eigenmodes in an indoor environment, so the peak power of BF delivered according to the targeted receive location also follows the spatial pattern of the eigenmodes. On the contrary, TR can focus waves in both space and time by utilizing multipath regardless of the targeted receive location and transmit array elements. As such, for each targeted location in an indoor environment, the received peak power from BF fluctuates according to the eigenmodes while TR has a consistently focused peak power, resulting in *PRPR* varying with the targeted location rather than the array element. As mentioned previously, although all array elements have similar *PRPR* values at each targeted location, the performance of TR-based WPT can be further optimized by using only the element with the largest *PRPR*.

Moreover, in terms of the time duration *T*, the impulse response in an indoor environment has a longer *T*, contributing to a higher *PRPR* value, and the impulse response in free space has a shorter *T*, contributing to a lower *PRPR* value. Both the free space and indoor results validate Eq. (7), that the level of *PRPR* can be obtained simply through the transfer function and beacon signal, and the performance of linear array-based WPT via BF and TR can be predicted according to the complexity of a given propagation environment. In the following subsection, the feasibility of *PRPR* is specifically discussed through comparison with the measured power transmission results.

### Power transmission results

**Figure 5:**
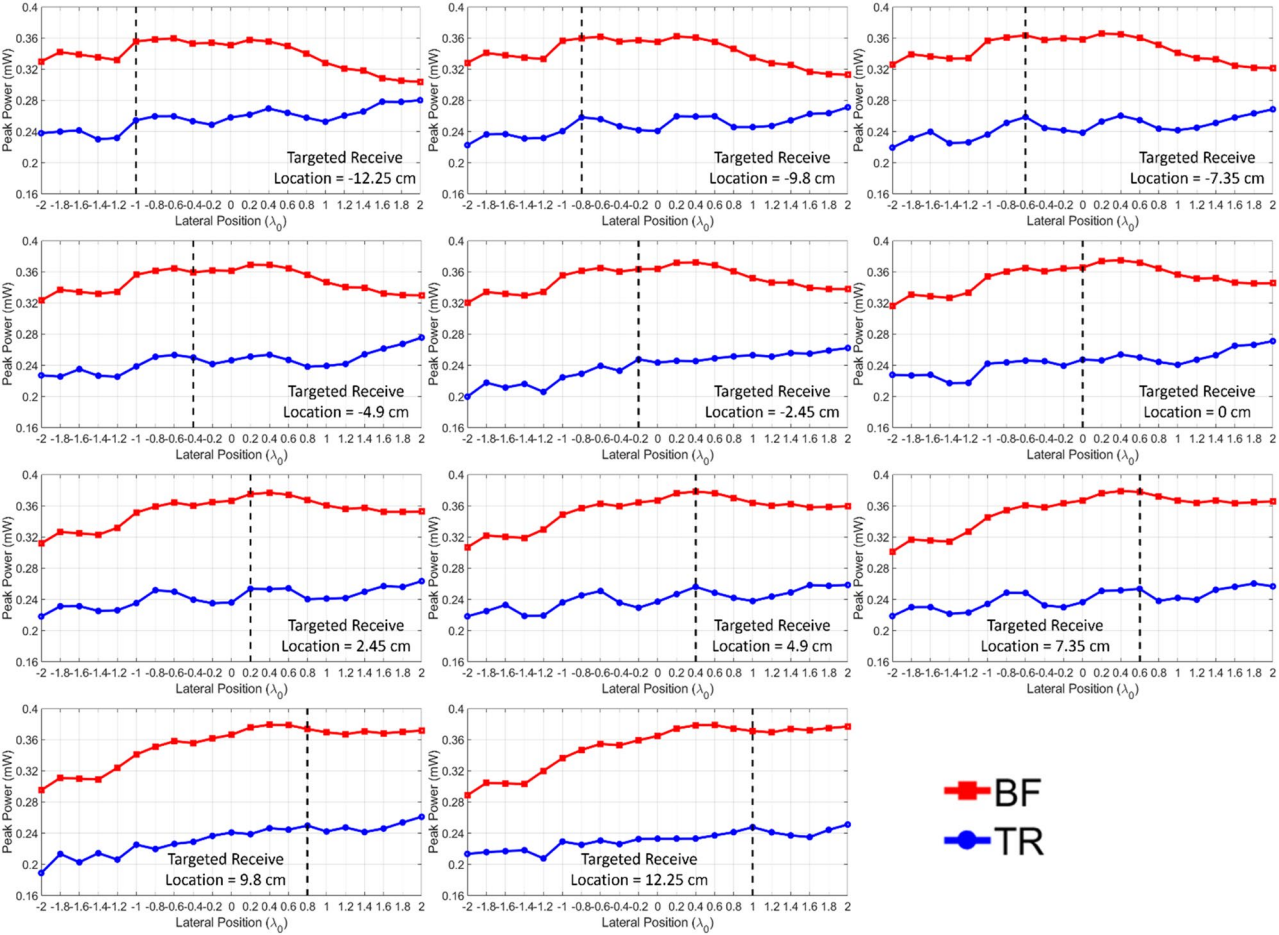
Measured peak power at the receiver as a function of lateral position resulting from BF and TR in free space.

As mentioned previously, both BF and TR are carried out in the processing by setting the same average transmit power, and the peak power of the receive signals is scanned over a lateral range of $\pm 2\lambda$. Here, the peak power is calculated based on a load impedance of 50 $\Omega$ on the receiving end. Note that for the TR processing, only one array element with the largest *PRPR* value is used. In Fig. 5, the peak power delivered by BF and TR as a function of the lateral position is plotted for all 11 cases of targeted receive locations for the case of free space. In the figure, the results indicate that the BF delivers higher peak power than TR at all 11 targeted receive antenna locations, in accordance with the *PRPR* values less than 1 for free space. In particular, the spatial pattern resulting from BF appears as a shape of a beam around each targeted receive location, while TR exhibits nearly consistent spatial pattern for all targeted receive locations. This can be explained by the fact that since there is no multipath in free space, the intended beam can be properly generated by BF, whereas TR loses its ability of wave focusing.

**Figure 6:**
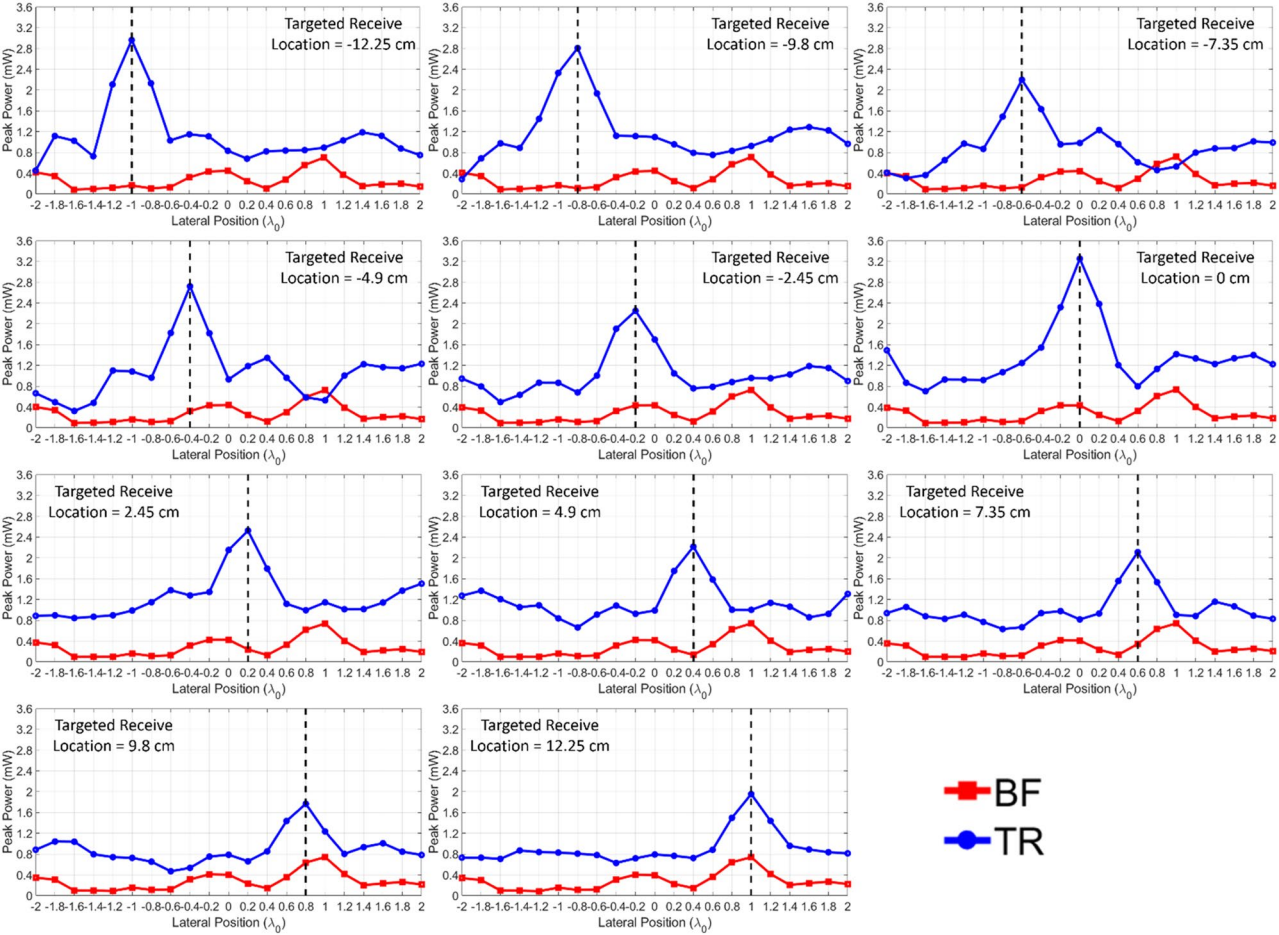
Measured peak power at the receiver as a function of lateral position resulting from BF and TR in an indoor environment.

On the other hand, for the case of an indoor multipath environment, TR delivers higher peak power compared to BF at all 11 targeted receive locations, which agrees with the *PRPR* results greater than 1, as shown in Fig. 6. Since the scatterers and reflectors cause multipath, TR can take advantage of them to selectively focus waves at desired locations, but BF is no longer able to selectively transmit due to the impaired beam. Hence, as shown in Fig. 6, the spatial pattern resulting from TR contains a distinct peak that consistently occurs at the desired receive location, but the spatial pattern resulting from BF remains nearly identical for all 11 targeted locations. As mentioned previously, the spatial pattern resulting from BF is dominated by the eigenmodes of the environment, and the modal behavior of the fields affected by these eigenmodes can be observed with relative peaks and nulls taking place. From this point of view, *PRPR* increases relatively further when the spatial pattern of BF corresponds to a null, and *PRPR* decreases relatively when the spatial pattern of BF corresponds to a peak.

From these power transmission results, the theoretical *PRPR* (calculated using Eq. (7)) and the experimental *PRPR* (calculated from measured peak power) are compared for each targeted receive location, and their maximum values with the corresponding antenna are shown in Table 1. The results in general show a good agreement between the theoretical *PRPR* and experimental *PRPR*, with a slight discrepancy in their values. This discrepancy is possibly due to the fact that in each case, *PRPR* is calculated in different domains. That is, the theoretical *PRPR* is calculated in the frequency-domain while the experimental *PRPR* is calculated from the time-domain peak power. Nevertheless, similar *PRPR* values from the same antenna in both cases demonstrate the effectiveness of the *PRPR* as a performance predictor for BF versus TR.

**Table 1:** Comparison of theoretical and experimental maximum *PRPR* values for each targeted received location.

Targeted receive location (cm from the reference position)	Free space		Indoor environment	
	Theoretical maximum *PRPR*	Experimental maximum *PRPR*	Theoretical maximum *PRPR*	Experimental maximum *PRPR*
− 12.25	0.7249 (Ant. D)	0.7153 (Ant. D)	16.9826 (Ant. D)	18.1424 (Ant. D)
− 9.8	0.7268 (Ant. D)	0.7178 (Ant. D)	26.7084 (Ant. D)	25.7037 (Ant. D)
− 7.35	0.7205 (Ant. D)	0.7116 (Ant. D)	16.7501 (Ant. D)	16.8763 (Ant. D)
− 4.9	0.7044 (Ant. D)	0.6959 (Ant. D)	8.1589 (Ant. D)	8.4969 (Ant. D)
− 2.45	0.6896 (Ant. C)	0.6818 (Ant. C)	4.8548 (Ant. A)	5.2236 (Ant. A)
0	0.6849 (Ant. D)	0.6768 (Ant. D)	7.2974 (Ant. A)	7.574 (Ant. A)
2.45	0.6838 (Ant. D)	0.6759 (Ant. D)	9.8459 (Ant. C)	10.5915 (Ant. C)
4.9	0.6849 (Ant. D)	0.6771 (Ant. D)	14.861 (Ant. B)	16.5608 (Ant. B)
7.35	0.6788 (Ant. D)	0.671 (Ant. D)	5.9349 (Ant. D)	6.1387 (Ant. D)
9.8	0.6761 (Ant. C)	0.6683 (Ant. C)	2.7848 (Ant. D)	2.7757 (Ant. D)
12.25	0.6749 (Ant. B)	0.6676 (Ant. B)	2.6717 (Ant. C)	2.6423 (Ant. C)

The overall results clearly show the validity of *PRPR* as a performance predictor and the correlation between *PRPR* and WPT performance. In a propagation environment with low complexity such as free space, BF can outperform TR since it can properly generate the intended beam around the targeted location, resulting in a small *PRPR* (< 1). However, in a propagation environment with high complexity such as an indoor environment, the selective high peak power due to the spatial and temporal wave focusing of TR leads to a large *PRPR* (≫ 1). Therefore, it can be concluded that *PRPR* makes it possible to predict the performance of BF versus TR simply by using the transfer functions, and one can implement it as a performance metric for deciding between BF and TR for optimal linear array-based WPT in a given propagation environment.

### WPT mode decision system using *PRPR*

**Figure 7:**
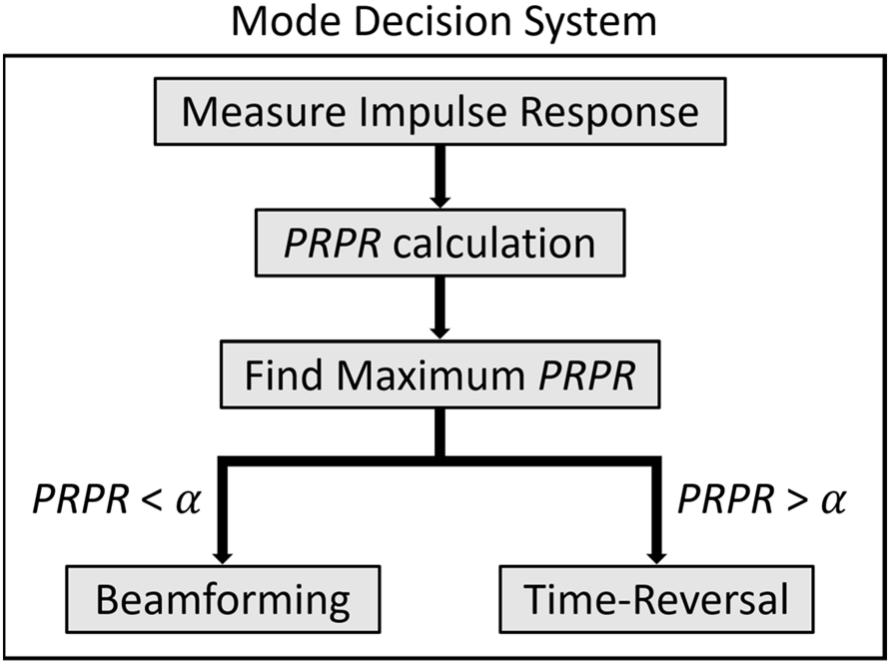
Flow chart of a notional mode decision system.

A notional mode decision system for linear array-based WPT is illustrated in Fig. 7. Here the WPT system is assumed to include both BF and TR transmitters. The process of WPT mode decision utilizing *PRPR* is as follows.

In a given propagation environment, the transfer functions between the transmit array and the receiver should first be measured. Next, using the measured transfer functions, *PRPR* corresponding to each array element can be calculated using Eq. (7). Here, it is possible to improve the performance of TR by choosing an array element that provides the largest value among the calculated *PRPR*s. After finding the maximum *PRPR*, this *PRPR* is compared to a reference value $\alpha$. This reference value would be set to 1 by default, but it may be modified to a different desired value depending on the array configuration and propagation environment. Consequently, by selecting TR-based WPT when *PRPR* is greater than $\alpha$ and BF when *PRPR* is less than $\alpha$, the mode decision of linear array-based WPT can be achieved. Considering that the performance of BF and TR varies depending on the complexity of the propagation environment, *PRPR* seems to be a key factor for WPT mode decision as well as a great performance metric for linear array-based WPT based on BF and TR. Therefore, a linear array-based WPT system capable of using both BF and TR with a mode decision system based on the concept of *PRPR* is a promising choice for efficient far-field WPT even in a dynamic propagation environment. Further theoretical development of *PRPR* for two- and three-dimensional transmit arrays can expand the scope of *PRPR*.

## Conclusion

In conclusion, a novel performance metric for linear array-based WPT via BF and TR defined in terms of *PRPR* was proposed and evaluated. By simply using the transfer function measured in a given propagation environment, the performance prediction of BF- and TR-based WPT is achieved with *PRPR*. The overall results demonstrate that in a low-complexity environment (i.e. free space), *PRPR* is less than 1 due to the high peak power by BF and short *T*, while in a highly complex environment, *PRPR* is greater than 1 due to the selective high peak power delivered by TR and long *T*. The results imply that the value of *PRPR* inherently exhibits qualitative information about the propagation environment, and therefore can serve as a great performance predictor between BF and TR. In a WPT system that can utilize both BF and TR, the concept of *PRPR* would be a deciding factor for mode selection between BF and TR for optimal wireless power transmission.
